# Perinatal use of triptans and other drugs for migraine—A nationwide drug utilization study

**DOI:** 10.1371/journal.pone.0256214

**Published:** 2021-08-23

**Authors:** Fatima Tauqeer, Mollie Wood, Sarah Hjorth, Angela Lupattelli, Hedvig Nordeng

**Affiliations:** 1 Pharmaco Epidemiology and Drug Safety Research Group, Department of Pharmacy, PharmaTox Strategic Research Initiative, Faculty of Mathematics and Natural Sciences, University of Oslo, Oslo, Norway; 2 Department of Epidemiology, T.H. Chan School of Public Health, Harvard University, Boston, MA, United States of America; 3 Department of Child Health and Development, Norwegian Institute of Public Health, Oslo, Norway; Universita degli Studi di Milano-Bicocca Scuola di Medicina e Chirurgia, ITALY

## Abstract

**Objective:**

To characterize nationwide utilization patterns of migraine pharmacotherapy before, during, and after pregnancy in women with triptan use.

**Methods:**

Population-based data were obtained by linking the Medical Birth Registry of Norway and the Norwegian Prescription Database from 2006 to 2017. We included 22,940 pregnancies among 19,669 women with at least one filled triptan prescription, a proxy for migraine, in the year before pregnancy or during pregnancy. The population was classified into four groups: i) continuers; ii) discontinuers; iii) initiators, and vi) post-partum re-initiators. Participant characteristics and prescription fills for other drugs such as analgesics, antinauseants, and preventive drugs among the groups were examined, along with an array of triptan utilization parameters.

**Results:**

In total, 20.0% of the women were classified as triptan continuers, 54.1% as discontinuers, 8.0% as initiators, and 17.6% as re-initiators. Extended use of triptans (≥15 daily drug doses/month) occurred among 6.9% of the continuers in the first trimester. The top 10% of triptan continuers and initiators accounted for 41% (95% CI: 39.2% - 42.5%) and 33% (95% CI: 30.3% - 35.8%) of the triptan volume, respectively. Triptan continuers and initiators had similar patterns of acute co-medication during pregnancy, but use of preventive drugs was more common among the continuers before, during, and after pregnancy.

**Conclusion:**

Among women using triptans before and during pregnancy, one in four continued triptan treatment during pregnancy, and extended triptan use was relatively low. Triptan discontinuation during and in the year after pregnancy was common. Use of other acute migraine treatments was higher among both continuers and initiators of triptans. Women using preventive migraine treatment were most commonly triptan continuers and re-initiators after pregnancy. Prescribing to and counseling of women with migraine should be tailored to the condition severity and their information needs to promote optimal migraine management in pregnancy.

## Introduction

Migraine is the fourth leading cause of years lived with disability among women of all ages, affecting up to 40% of women of reproductive age [1–3]. Although migraine symptoms improve in one-half to three-fourths of pregnant women, symptoms recur in up to 55% within one month postpartum [4], and up to 8% of women experience migraine throughout pregnancy [5]. Women with untreated migraine are at greater risk of several adverse pregnancy outcomes including preeclampsia, premature delivery, and low birth weight infants [6–8]. Almost two out of three women with migraine seek clinical help for their condition during pregnancy [9].

Pharmacotherapy is commonly prescribed to prevent or manage migraine in women of childbearing age [10–15]. The continuation of the treatment in pregnancy is sometimes needed to ensure optimal antenatal care [10, 16]. This therapy is preferably acute and given in combination with preventive drugs in patients with more than two migraine attacks per month [12, 13]. Acute pharmacological treatment ranges from over-the-counter analgesics such as nonsteroidal anti-inflammatory drugs (NSAIDs) to triptans and opioids [16–18]. Preventive migraine treatment with, for example, beta-blockers, tricyclic antidepressants, or antiepileptics, is generally not recommended in pregnancy but might be needed in some cases [16]. Ailments commonly associated with migrainous attacks, such as nausea and vomiting, may necessitate use of adjunctive antinauseants in pregnancy [12, 19]. Additionally, newer preventive treatments with monoclonal antibodies are available, including calcitonin gene-related peptides [20]. The availability of multiple treatment options and the elevated perceived risk of drug use in the perinatal period make management of migraine a challenge for both pregnant women and their prescribers [16]. Most studies of associations between migraine and birth/pregnancy/infant outcomes have not assessed the role of treatment, and available evidence does not suggest that triptan use in pregnancy appreciably alters the risk but migraine treatment is heterogeneous and more research is needed.

Drug utilization studies may enhance our understanding of drug use in the real world and consequently improve advice on management for complex diseases like migraine and comorbidities in pregnancy. A small number of studies have involved investigation of drug use among pregnant women with migraine [21], focusing predominantly on triptans [6, 22–24]. One Norwegian study of women with migraine (n = 401) found that 73.3% used migraine pharmacotherapy during pregnancy, and in this group, triptans (71.1%), paracetamol (63.1%), and NSAIDs (60.1%) were the most common agents [25]. Other studies have shown that pregnant women with migraine often avoid triptans and have a high likelihood of switching to other pain drugs such as NSAIDs and paracetamol [18, 26, 27], but little is known about patterns of perinatal use of multiple drugs for migraine in women with moderate to severe migraine [10].

In this study, therefore, we sought to characterize nationwide use patterns of migraine pharmacotherapy before, during, and after pregnancy among women in Norway, covering the years 2006 to 2017. We used filled prescriptions as a proxy for moderate to severe migraine. The specific aims were to examine triptan use patterns in the year before pregnancy and during and within one year after pregnancy, and to examine and quantify polytherapy (use of analgesics, antinauseants, and preventive drugs) by triptan exposure status during the perinatal period.

## Materials and methods

### Study design and data sources

For this descriptive population-based study, using unique personal identification numbers given to all residents of Norway, we linked two Norwegian nationwide health registries: the Medical Birth Register of Norway (MBRN) [28], and the Norwegian Prescription Database (NorPD) [29]. The MBRN contains data from compulsory medical records on all live births, stillbirths, miscarriages, and induced abortions after gestational week 12 [28]. It also has information on gestational age at delivery, maternal pre-existing conditions, age, and other demographics. Notification to MBRN is compulsory and is provided by midwives and physicians attending the birth using a standardised form. The forms with specific checkboxes on several chronic diseases such as epilepsy, chronic hypertension, diabetes, asthma and chronic kidney disease to ensure that these are adequately captured. The clinical diagnoses were registered according to the International Classification of Diagnoses (ICD) by the ICD-10 codes from 1999 and onwards. The NorPD contains records on all prescriptions, regardless of reimbursement, dispensed in ambulatory care since 2004 [29]. All dispensed drugs are categorized according to the Anatomic Therapeutic Chemical (ATC) classification system established by the World Health Organization [30].

### Study population

**Fig 1:**
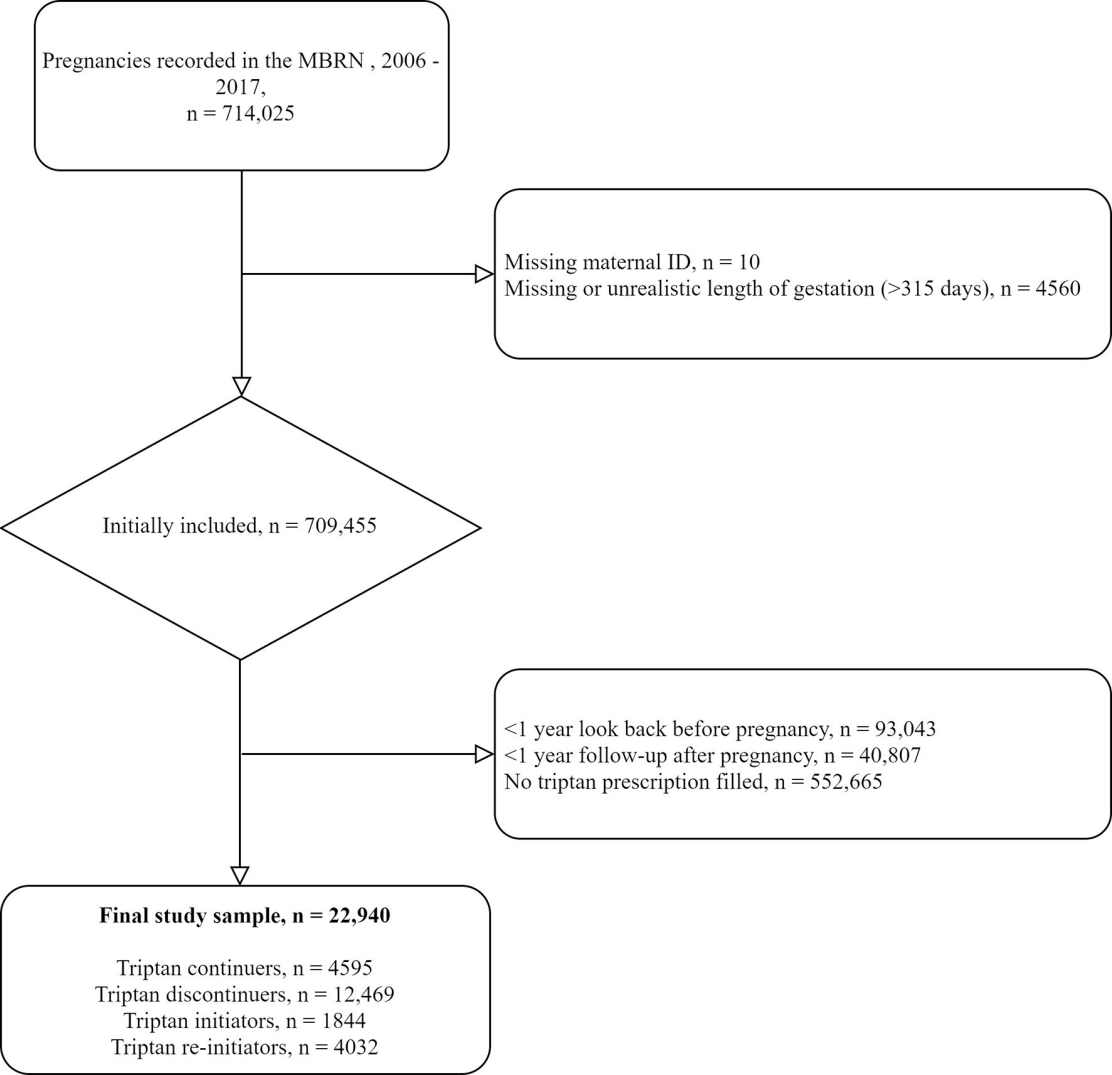
Flowchart of study inclusions and exclusions.

From the MBRN, we identified all pregnancies (n = 714,025) from January 1, 2006, to December 31, 2017, that led to birth after gestational week 12. NorPD is considered incomplete during 2004, the first year after the registration started [31], so to allow one year of look-back from pregnancy start, we included pregnancies that began no earlier than January 1, 2006. Fig 1 outlines the exclusion criteria that led to the final number included in the study (Fig 1). Because maternal migraine diagnostic codes are not available in NorPD or MBRN, we used triptan prescriptions as a proxy for maternal migraine. Specifically, we classified women as having migraine if they filled one or more prescriptions for a triptan (ATC-code N02CC) at any point between one year before pregnancy start through the end of pregnancy. Our final dataset represented 22,940 pregnancies and 19,669 women.

### Definition of triptan exposure

All filled triptan prescriptions were identified from the NorPD. We defined exposure to triptans and other drugs according to the ATC classification and studied three exposure periods: one year before pregnancy start, during pregnancy, and one year after the end of pregnancy. To calculate the date of pregnancy start, we subtracted the gestation length in days from the date of birth, as ascertained from the MBRN. Trimesters were defined as first (T1 = days 0–90 since pregnancy start), second (T2 = days 91–180), and third (T3 = 181 days until pregnancy end). To account for drug supply that overlapped with these exposure periods, we used information about the amount dispensed as defined daily doses (DDDs). For each period, we identified the proportion of pregnancies associated with a redeemed prescription of triptan.

In a population of women with a proxy for migraine within the period from one year before to the end of pregnancy, we defined four mutually exclusive groups (S1 Fig in S1 File). Group 1 consisted of triptan continuers, the pregnancies in which women had filled a prescription before pregnancy and continued to do so during pregnancy. Triptan discontinuers formed group 2, defined as pregnancies in which women had filled a triptan prescription before pregnancy but did not do so during pregnancy or at one year after the pregnancy ended. Group 3 consisted of triptan initiators, all pregnancies in which women began filling triptan prescriptions during pregnancy and had no history of triptan prescriptions filled in the one-year period before pregnancy. The final group included the triptan re-initiators, defined as pregnancies in which women discontinued triptans during pregnancy and were dispensed triptans again within one year after the end of the pregnancy.

### Triptan drug utilization measures

We quantified the extended use of triptans, defined as ≥15 DDDs/month, selecting this threshold primarily based on the International Classification of Headache Disorders [32]. The DDD is defined as the assumed average maintenance dose of triptan per day for treatment of migraine in adults, and varies according to the individual triptan and its administration form. For example, the DDD for nasal sumatriptan corresponds to 20 mg, thus the extended use of sumatriptan nasal spray corresponds to use of 20 mg of sumatriptan nasal spray for 15 days per month or more. DDDs for each triptan according to the route of administration are presented in the supplementary (S2 Table in S1 File).

The definition of extended triptan use was applied to the pre-pregnancy, pregnancy, and post-pregnancy periods, individually. We also estimated the median of cumulative DDDs and mean number of prescriptions before, during, and one year after the end of each pregnancy.

### Other migraine treatment

We defined several other groups of medications that may be used to treat migraine based on Norwegian guidelines on migraine and pregnancy [33] (designated here collectively as “other migraine medications”) as the filling of at least one prescription for the following: acute analgesics, including paracetamol, codeine and paracetamol combination, ibuprofen, diclofenac, naproxen, and tolfenamic acid; preventive migraine drugs, including metoprolol, propranolol amitriptyline, candesartan, topiramate, valproic acid, botulinum toxin, clonidine, lisinopril, and verapamil; and antinauseants, including metoclopramide, doxylamine, meclozine, prochlorperazine, promethazine, and serotonin antagonists. We attempted to capture calcitonin gene-related peptide antagonists in our study data but did not find any users primarily because these drugs were first marketed for migraine prophylaxis in Norway in 2018.

Medications were categorized by timing of prescription fill, occurring in the year before pregnancy, during pregnancy, and within one year after pregnancy. DDDs dispensed before pregnancy but overlapping with the pregnancy start and those within the pregnancy window were counted as continuously used drugs only because these are taken more chronically (S2 Table in S1 File). Some of the studied drugs were also available over the counter (S2 Table in S1 File). Several of the drugs were not licensed for migraine treatment but were commonly used in clinical practice (S2 Table in S1 File) [15, 33].

### Migraine severity

We classified migraine severity based on the type and timing of migraine drugs dispensed and the route of administration (Table 1). This classification relied on current pharmacotherapeutic guidelines and treatment algorithms [5, 12, 13, 15, 16, 34]. Because triptan use was an inclusion criterion to identify women with migraine, women with mild migraine who achieved sufficient effect using paracetamol and/or NSAIDs were by default not included in the study (Table 1).

**Table 1:** Classification of severity according to prescription drug treatment*.

Migraine severity	Definition
Mild	Sufficient effect using paracetamol and/or NSAIDs only, and no triptan use **
Moderate	Use of triptans before and/or during pregnancy (excluding sumatriptan injection)
Severe	Sumatriptan injection before and/or during pregnancy and/or migraine prophylaxis before but not during pregnancy
Very severe	Migraine prophylaxis during pregnancy

*Algorithm is based on national clinical guidelines for the treatment of women with migraine in pregnancy [5, 12, 13, 15, 16, 31].

** By default these women were not included in this study because filling a triptan prescription before and during pregnancy was used to identify the study population.

### Maternal characteristics

Maternal characteristics considered included age, marital status, employment, parity, chronic illness, weight gain in pregnancy, and smoking status before and at the end of pregnancy, as ascertained in the MBRN. We computed an obstetric comorbidity index, adapted from Bateman et al. [35], using the following variables available in the MBRN: age, asthma, pre-gestational diabetes, chronic hypertension, gestational hypertension, kidney disease, previous caesarean section, multiple gestation, and preeclampsia. These factors were weighted as recommended by Bateman et al. [35]. Scores ranged from 0 to 12, with higher scores indicating higher obstetric comorbidity.

### Data analysis

First, we described maternal characteristics across the four triptan exposure groups and the pattern of use of different types of triptans by way of continuers, discontinuers, initiators, and re-initiators. For this purpose, we plotted Lorenz curves with 95% confidence intervals to describe the skewness in drug use and calculated the Gini coefficient to identify excessive triptan users, as measured in DDDs dispensed [36]. A straight diagonal line for the Lorenz curve indicates an equal distribution of doses among the users. The Gini coefficient is a measure of the inequality seen in the Lorenz curve, with a value of 0 expressing total equality and a value of 1 indicating the highest inequality [36].

Co-medication was determined by calculating and plotting the proportion of cases in which other drugs were used, such as analgesics, antinauseants, and preventive medication pre-pregnancy, during pregnancy, and one year after the end of pregnancy.

Potential heterogeneity in the indication for treatment with preventive migraine drugs could result in different patterns of prescription fills. For this reason, we conducted a sensitivity analysis by excluding women with epilepsy or chronic hypertension, recorded in the MBRN before the pregnancy (n = 461).

To assess the association between triptan groups and the migraine severity algorithm, we used a chi-square test accounting for clustered data as some women participated in the study with more than one pregnancy [37]. We also performed a sub-analysis using the severity of migraine to determine co-medication use. In a *post hoc* sensitivity analysis to assess the impact of including multiple pregnancies for some women, we restricted the sample to one random pregnancy per woman. For all statistical analyses, we used Stata, version 16.

### Ethics

The Regional Committee for Research Ethics in South-Eastern Norway (approval number 2018/140/REK Sør Øst) and the Data Protection Officer at the University of Oslo (approval number 58033) approved this study, in compliance with the General Data Protection Regulation. Data were stored at the TSD (Tjeneste for Sensitive Data, p704) facilities, owned by the University of Oslo and operated and developed by the TSD service group at the University of Oslo, IT-Department.

## Results

Of the 22,940 included pregnancies among 19,669 women with migraine, we identified 4595 (20.0%) triptan continuers in pregnancy, 12,469 (54.1%) triptan discontinuers, 1844 (8.0%) triptan initiators in pregnancy, and 4032 (17.6%) triptan re-initiators by one year after pregnancy. Triptan continuers and re-initiators were slightly older and had a higher obstetric comorbidity index compared with the discontinuers and the initiators. The initiators were more often unemployed. The discontinuers were more often nulliparous, whereas the initiators and re-initiators were more often parous and had more often experienced a previous pregnancy loss. There was a higher proportion of smokers before pregnancy and at the end of pregnancy among the initiators (Table 2).

**Table 2:** Characteristics of the included women (n = 22,940), grouped by exposures, 2006–2017.

Characteristics	Triptan continuers	Triptan discontinuers	Triptan initiators	Triptan re-initiators
	n = 4595 (%)	n = 12,469 (%)	n = 1844 (%)	n = 4032 (%)
Maternal age, mean (SD)	31.2 (5.2)	29.8 (5.2)	29.1 (5.4)	30.7 (5.2)
Married/cohabiting (yes)	4221 (91.9)	11494 (92.2)	1671 (90.6)	3753 (93.1)
Employed (yes)	3164 (68.9)	8563 (68.7)	1242 (67.3)	2782 (69.0)
Gestational length,				
Days, mean (SD)	276.1 ±18.9	276.6 ±17.8	277.1 ±17.5	275.0 ±20.7
Weeks, mean (SD)	39.0 ±2.7	39.1 ±2.6	39.2 ±2.5	38.9 ±3.0
Parity				
0	1935 (42.1)	5678 (45.5)	736 (39.9)	1672 (41.5)
1+	2660 (57.9)	6791 (54.5)	1108 (60.1)	2360 (58.5)
Previous pregnancy loss (yes)	973 (21.2)	2682 (21.5)	421 (22.8)	914 (22.7)
Obstetric comorbidity index‡, mean (SD)	0.62 (1.1)	0.51 (1.0)	0.51 (1.0)	0.65 (1.1)
*Components of the index*				
Asthma	278 (6.1)	898 (7.2)	163 (8.8)	280 (6.9)
Diabetes, pre-gestational	45 (1.0)	88 (0.7)	20 (1.1)	35 (0.9)
Chronic hypertension	68 (1.5)	149 (1.2)	16 (0.9)	63 (1.6)
Gestational hypertension	133 (2.9)	316 (2.5)	50 (2.7)	102 (2.5)
Kidney disease	36 (0.8)	109 (0.9)	22 (1.2)	36 (0.9)
Multiple gestation	79 (1.7)	195 (1.6)	27 (1.5)	117 (2.9)
Preeclampsia, mild	112 (2.4)	291 (2.3)	37 (2.0)	114 (2.8)
Preeclampsia, severe	92 (2.0)	187 (1.5)	23 (1.3)	70 (1.7)
Previous caesarean section	312 (6.8)	759 (6.1)	137 (7.4)	321 (8.0)
Epilepsy	37 (0.8)	92 (0.7)	15 (0.8)	24 (0.6)
Rheumatoid arthritis	44 (1.0)	71 (0.6)	9 (0.5)	21 (0.5)
Weight gain in pregnancy (kg), mean (SD)	13.9 (7.0)	14.1 (7.7)	13.8 (6.5)	14.4 (7.7)
Smoking before pregnancy (yes)	442 (9.6)	1148 (9.2)	230 (12.5)	403 (10.0)
Smoking, end of pregnancy (yes)	266 (5.8)	741 (5.9)	143 (7.8)	243 (6.0)

Data are expressed as n (%) unless otherwise stated. Missing values ranged from 0% (maternal age, parity) to 17.7% (maternal employment).

For maternal smoking, the women could choose not to have the information reported to the registries; up to 12.4% of women chose this.

‡ The scores ranged from 0 to 12 in the study sample, with higher scores indicating higher obstetric comorbidity. Adapted from Bateman et al. [19] using the variables available in MBRN (age, asthma, pre-gestational diabetes, chronic hypertension, kidney disease, previous caesarean section, multiple gestation, severe preeclampsia, mild preeclampsia and gestational hypertension) and weighting the variables as done by Bateman et al.

### Patterns of triptan use

**Fig 2:**
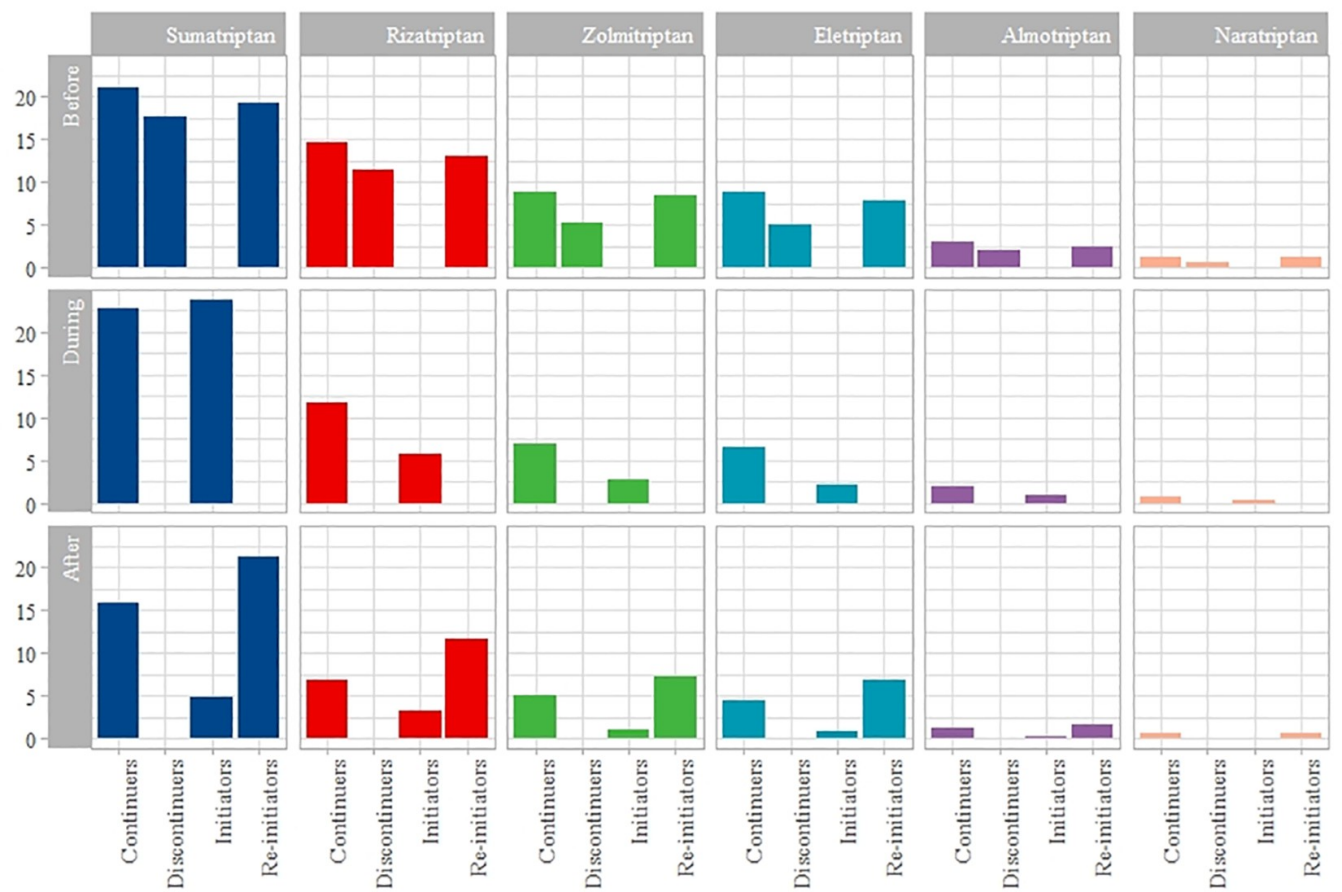
Proportion of pregnancies with filled prescriptions for different triptans according to timing in pregnancy and triptan utilization group (n = 22,940). Sum of percentages is greater than 100% because some women used several types of triptans. Before = one year before pregnancy, during = during pregnancy, after = one year after pregnancy.

Fig 2 displays the different triptans used among the four exposure groups before, during, and after pregnancy during 2006–2017. Sumatriptan was the most common triptan filled at each time period, followed by rizatriptan and zolmitriptan. Among the continuers, the sumatriptan prescription fills increased slightly throughout pregnancy (22.7%) and declined after pregnancy (15.8%), but not to pre-pregnancy levels (21.0%). The initiators filled the highest proportion of sumatriptan prescriptions during pregnancy (23.7%), followed by a rapid decline after pregnancy (4.9%). The proportions were similar before (19.3%) and after (21.2%) pregnancy for re-initiators (Fig 2). The continuers used a higher proportion of other triptans during pregnancy than the initiators. The re-initiators used the highest proportion of triptans after pregnancy.

As presented in Table 3, during pregnancy, extended use of triptans occurred in 6.9% of the continuers in T1. They had higher median DDDs than initiators during pregnancy (18 vs 6 DDDs). We also found a higher mean number of triptan prescriptions filled in pregnancy among the continuers (1.9 prescriptions) compared with the initiators (1.3 prescriptions). In the post-pregnancy period, the continuers had the highest extended use of triptans (5.8%) and median DDDs (36 DDDs per year). The mean number of prescriptions for continuers and re-initiators did not differ substantially.

**Table 3:** Drug utilization parameters for triptan use among exposure groups during and after pregnancy, 2006–2017 (n = 22,940).

Parameters	Discontinuers	Continuers		Initiators	Re-initiators			
	Pre	Pre	During	Post	During	Post	Pre	Post
**Extended use of triptans** * **, n (%)**	96 (0.8)	554 (12.1)	T1: 318 (6.9)	265 (5.8)	T1: 12 (0.7)	<5	151 (3.7)	55 (1.4)
			T2: 125 (2.7)		T2: 6 (0.3)			
			T3: 71 (1.6)		T3: 8 (0.4)			
**Cumulative DDDs, median (IQR)**	12 (6–24)	48 (18–105)	T1: 18 (6–24)	36 (18–90)	T1: 6 (6–12)	12 (6–24)	24 (12–54)	18 (12–36)
			T2: 18 (9–30)		T2: 6 (6–12)			
			T3: 18 (12–30)		T3: 6 (6–12)			
**Rx filled Mean (SD)**	1.9 (1.8)	5.5 (5.4)	1.9 (2.2)	2.6 (4.3)	1.3 (0.9)	0.5 (1.4)	3.4 (3.0)	2.2 (2.1)

Rx: prescription; pre: one year before pregnancy; post: one year after pregnancy; T1: first trimester; T2: second trimester; T3: third trimester; DDD: defined daily doses; IQR: interquartile range.

* Extended use of triptans defined as ≥15 DDDs/month.

**Fig 3:**
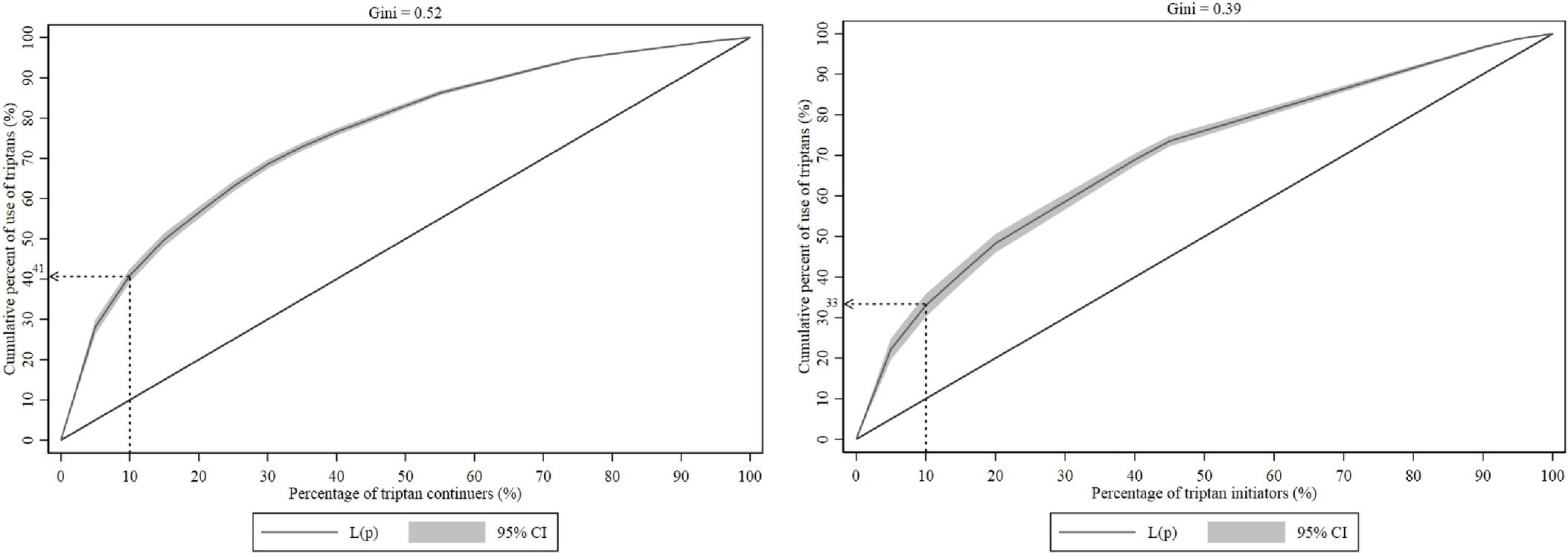
Lorenz curves for triptan users during pregnancy (continuers and initiators). The curved line indicates the proportion of triptan use accounted for by the proportion of the most intensive users. The top 10% of the users consumed 41% (95% CI: 39.2% - 42.5%) of the triptan volume among continuers and 33% (95% CI: 30.3% - 35.8%) among initiators. The Gini coefficients were 0.52 for the continuers and 0.39 for the initiators, indicating some inequality in triptan use.

The Lorenz curves for the use of triptans during pregnancy are shown in Fig 3. The Gini coefficients were 0.52 for the continuers group and 0.39 for the initiators group, indicating some inequality in triptan consumption. The top 10% of triptan continuers and initiators accounted for 41% (95% CI: 39.2% - 42.5%) and 33% (95% CI: 30.3% - 35.8%) of the triptan volume, respectively (Fig 3).

### Migraine severity pattern among the study sample

According to the migraine severity classification, 92.6% of the included cases were in the moderate category. In total, 5.2% of women had severe migraine, and 2.2% of women were categorized as having a very severe migraine, i.e., requiring first-, second-, or third-line migraine prophylaxis in pregnancy (Table 4). By cross tabulating the severity groups with the exposure groups, we found that the proportion of the continuers increased as the severity increased (18.7% moderate vs 42.8% severe).

**Table 4:** Classification of migraine severity according to the triptan exposure groups.

Exposure groups	Moderate migraine	Severe migraine	Very severe migraine
N = 22,940 (100%)	n = 21,242 (92.6%)	n = 1189 (5.2%)	n = 509 (2.2%)
Triptan continuers (20.0)	3973 (18.7)	404 (34.0)	218 (42.8)
Triptan discontinuers (54.3)	11,828(55.7)	477(40.1)	164 (32.2)
Triptan initiators (8.0)	1769 (8.3)	42 (3.5)	33 (6.5)
Triptan re-initiators (17.6)	3672 (17.3)	266 (22.4)	94 (18.5)
Total	21,242 (100.0)	1189 (100.0)	509 (100.0)

Please see Table 1 for the definition of migraine severity. Chi-square test accounting for clustering, i.e., one woman could participate with more than one pregnancy, P < 0.001.

### Use of other migraine treatments

**Fig 4:**
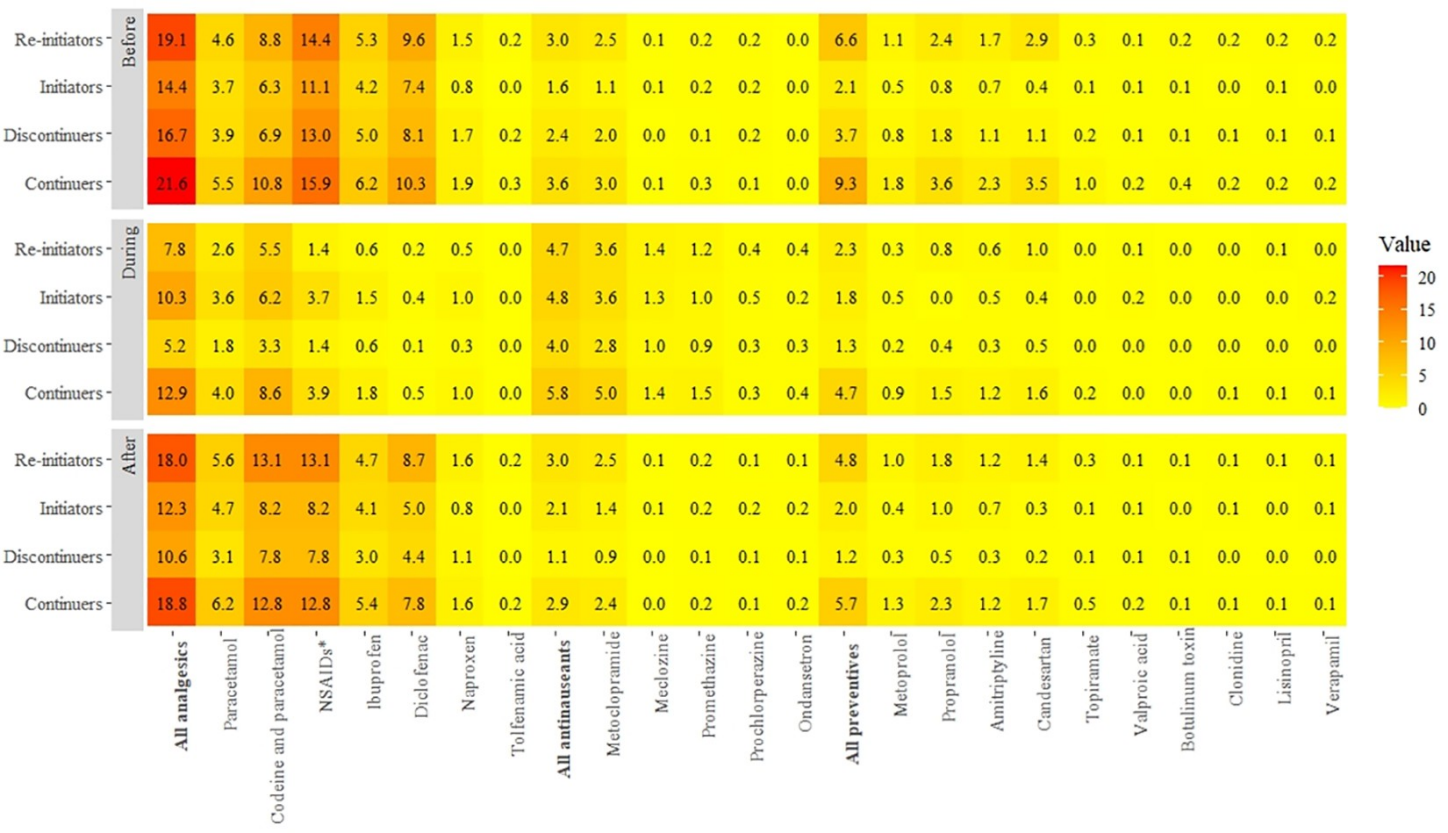
Heatmap illustrating the proportion of pregnancies with filled prescriptions for other drugs among exposure groups before, during, and after pregnancy, 2006–2017. The darkest color indicates the maximum proportion of prescription fills.

As shown in Fig 4, analgesics were the most-filled prescriptions before, during, and after pregnancy. During pregnancy, among the continuers, 12.9% used analgesics concomitantly with triptans, which was higher than the initiators (10.3%). Among the discontinuers, 5.2% used concomitant analgesics and 4.0% used antinauseants. Antinauseants were more frequently filled among the continuers (5.8%). Continuers and initiators showed the highest percentage of co-medication use with NSAIDs during pregnancy, and the highest percentage of preventive treatment use was identified among the continuers before (9.3%), during (4.7%), and after pregnancy (5.7%) (Fig 4). Extended use of triptans in pregnancy was more common among continuers on preventive treatment before pregnancy (n = 28/426; 6.6%) compared with continuers without preventive treatment before pregnancy (n = 78/4169; 1.9%).

In the sub-analysis, we observed that the use of other drugs such as analgesics and antinauseants in pregnancy was more common in the severe and very severe groups. The ‘very severe’ group used preventive drugs throughout the study period (before, during, and after pregnancy), but the proportion of prescription fills declined rapidly after pregnancy (38.9% vs 100%) (S3 Table in S1 File).

### Sensitivity analyses

Patterns of prescription fillings for other migraine treatments among the exposure groups were largely unchanged in the sensitivity analyses, from which we excluded 464 women with a history of epilepsy or chronic hypertension (S4 Table in S1 File). We also carried out a post-hoc sensitivity analysis including only one random pregnancy per woman to describe the utilization parameters of triptans. These tables are now included as supplementary results (S5 & S6 Tables in S1 File). These sensitivity analyses results align with the main results.

## Discussion

Approximately one in four women in this study filled at least one triptan prescription during pregnancy. However, half of the included women discontinued filling triptan prescriptions for the entire period of pregnancy and one year after pregnancy. A small number of triptan users consumed a majority of the triptan volume during pregnancy. We observed polytherapy with other acute drugs, such as analgesics and antinauseants, more often among continuers and initiators during pregnancy. Co-medication with preventive treatment was more common among continuers and re-initiators during pregnancy.

Our drug utilization findings show a decrease in triptan use during pregnancy compared with before and after pregnancy, consistent with previous studies [26, 27]. A reduction in triptan use during pregnancy might trace to several factors. First, health care providers and pregnant women with migraine prefer to minimize drug use during pregnancy, based on concerns regarding fetal safety [16, 38]. Second, 60% to 70% of women with migraine report an improvement during gestation [8, 16, 23], suggesting that some discontinuation in our study was likely attributable to the lower need for medication in pregnancy. Registry-based studies are not well suited to determine reasons for discontinuing medications, and future studies should also involve interviews and qualitative methods.

The most common triptan filled was sumatriptan, in line with previous research [22]. This result is not surprising considering that sumatriptan is the triptan that has been most extensively examined in pregnancy, without concerning findings [18]. However, the women in our study used other triptans during pregnancy and the post-pregnancy period. One possible reason may be that not all patients with migraine benefit from sumatriptan, and because triptans are the first-line treatment for migraine, they tried other oral triptans. This finding also raises concerns given the limited and somewhat conflicting safety data available to support the efficacy, tolerability, and safety of other triptans during the perinatal period [16, 18, 39]. Because of their lipophilic nature, these drugs are more likely to penetrate the placental barrier and are not preferred during pregnancy and lactation for this reason [3]. In light of these findings, we need to reconsider perinatal research and make it more patient-centered, such as focusing on the real-world effectiveness of triptans in pregnancy.

The use of several drug utilization metrics and various comparison groups of women with migraine revealed several interesting results. A median of 18 DDDs dispensed to continuers per trimester suggests frequent use of triptans. Almost 6.9% of the continuers had extended triptan use in T1, defined as more than 15 DDDs dispensed per month, a level of use that indicates high migraine severity among some pregnant women in the first trimester. We might be overestimating extended triptan use because the available literature defines triptan overuse in a range of ways [22, 40]. From a clinical perspective, however, this finding calls attention to the need for patient counseling because extended intake of triptans can lead to medication-overuse headache [41]. Among continuers, we found that extended use of triptan was more common in women with a history of preventive treatment, indicating a history of severe migraine. We suggest the use of migraine severity algorithms to capture complex intermittent/as-needed drug use patterns rather than focusing on individual drug groups.

The Lorenz curves offer further support for the findings from utilization metrics, indicating that a small number of women filled prescriptions for the majority of the triptan drug volume. The 10% of triptan users accounted for 41% of the DDDs among continuers and 33% among initiators, which are comparatively high values and suggest that some women are heavy users of triptans during and after pregnancy. The Gini coefficients of 0.52 (continuers) and 0.39 (initiators) also suggest an unequal distribution of triptan use during pregnancy. These results are consistent with those of a previous study on the use of sumatriptan among general sumatriptan users. Those authors found that a small proportion of the patients (1%) were using excessive amounts of sumatriptan (20%), highlighting inappropriate use of the drug [42]. It is worth noting that comparable data are scarce on triptan utilization metrics in pregnant women with migraine.

Our results suggest that a higher proportion of continuers and re-initiators belong to the severe to very severe category of migraine rather than to the moderate category. They more often used second- and third-line preventive drugs and had higher comorbidity index scores than the other two groups. Thus, healthcare providers must focus on rational and personalized pharmacotherapy among pregnant women with migraine and comorbidities. From a methodological perspective, these findings may suggest that triptan continuers and re-initiators are comparable in terms of disease severity in pregnancy. Such information can guide comparison group selection in future safety studies on migraine pharmacotherapy during pregnancy.

Using migraine severity algorithms revealed considerable triptan continuation during pregnancy and showed that women with severe and very severe migraine more often use other migraine treatments during the perinatal period. Findings from the table also illustrated how the disease progressed over the course of a pregnancy; for instance, a pregnancy might be a trigger for discontinuing preventive drugs in the severe category. Future studies of medication exposure–pregnancy outcome associations should evaluate whether triptan exposure, discontinuation groups, and severity algorithms give similar results.

Managing migraine in pregnancy is clinically challenging because each patient responds differently to available, acute-acting drugs. Therapeutic failure is a common issue that triggers switching to or concomitant use of other acute treatment options. Our findings show higher proportions of analgesics and antinauseants used in combination with triptans in continuers and initiators in pregnancy. This result may indicate their use not only to alleviate nausea during migraine attacks but also to treat nausea and vomiting in pregnancy [16]. Health care providers involved in the management of migraine should evaluate medication needs and efficiency in every trimester so that women with migraine are adequately treated during pregnancy and to avoid the risk of medication overuse. The findings of this study provide real world data on dispensing practices for migraine medication to pregnant women in Norway, and shows that these women have complex drug prescription regimens, and are at risk of medication overuse in the perinatal period. This knowledge can be used to remind prescribers of the importance of periconceptional counselling for women with migraine, and the need to closely monitor migraine severity, establish treatment goals and discuss benefits and risks of all medications used to treat migraine during pregnancy and the post-partum period.

### Strengths and limitations

Our study has several limitations. Filling a prescription is not equivalent to actual drug use. This is especially true for triptans, which may be prescribed days or weeks before a migraine attack occurs. We also had no information on prescribed doses, adherence to prescribed dosages, or timing of when the drugs were taken, which could have resulted in misclassification of the groups. Furthermore, because we focused on women receiving care for migraine, we might have underrepresented migraine by excluding women with less severe migraine who did not use a triptan or women with contraindications for triptan use. According to one study that measured agreement between dispensed and used triptans in pregnancy, use of prescription filling rather than self-report to define exposure to triptans during pregnancy could result in substantial underestimation of exposure (sensitivity: 39.1%, specificity: 95.4%). Therefore, exposure misclassification cannot be ignored in the current study [43]. In addition, our study population may have included women with cluster headache. However, because cluster headache is very rare (less than 1 in 1000 adults) compared to migraine (1 in 9 adults) [44], the possible impact on our estimates is likely to be very low. In our study, women could participate with more than one pregnancy; the sensitivity analysis including one randomly-selected pregnancy per woman did not deviate from the main results.

In previous studies, migraine has been defined using diagnostic codes from health care registries [45]. In the current work, we used filling a triptan prescription as a proxy for migraine diagnosis. A similar approach was adopted by the authors of an Italian study, who observed a burden of unmet medical needs among migraine patients treated with triptans [40]. We did not have data on diagnosis (e.g., ICD-10 codes), subtypes, or severity of migraine but relied on a classification based on available literature and treatment guidelines. This classification, however, has not been validated. Through this classification, we might have overestimated or underestimated the numbers in each severity category, including women who used a drug but did not need it or excluding women who did not use a drug but needed it. Furthermore, extended use of triptan in the year before and after pregnancy may be greater than during pregnancy because women had a greater opportunity (longer time period) to overuse the drug. Finally, the NorPD does not include over-the-counter drugs and drugs dispensed in hospitals, so percentages of drug use will be underestimated, especially in the case of analgesics and antinauseants.

This study also has several strengths. Taking advantage of the 15-year period covered by NorPD and the population-based nature of the MBRN, it is the first nationwide study in Norway to map longitudinal patterns of triptan use from one year before pregnancy to one year after the end of pregnancy. This study is novel in providing new insights about women who initiated triptan treatment specifically during pregnancy, as well as those who paused pharmacotherapy during pregnancy. Moreover, our findings provide new knowledge about the extent of migraine co-medication among triptan discontinuers, continuers, initiators, and re-initiators. We implemented multiple approaches and an array of utilization metrics to describe pattern of triptans and the extent of other migraine drug use during and after pregnancy. Our attempt to classify migraine severity by way of drug use is novel and might find applications in subsequent registry-based studies of migraine.

## Conclusion

In this large, population-based drug utilization study, we found that most women who use triptans before pregnancy discontinue these drugs during pregnancy. Use of other acute migraine treatment is greater among both continuers and initiators of triptans. Women using preventive migraine treatment were most commonly triptan continuers and re-initiators after pregnancy. Despite being rare, this finding of women with extended triptan use during pregnancy raises concerns. Differences in migraine severity and willingness to use migraine drugs during pregnancy may explain variations in drug utilization patterns among different migraine groups. Management of migraine among women of childbearing age should be tailored according to migraine severity, pregnancy status, and the patient’s information needs.

## Supporting information

S1 File (DOCX)
