# Supplementary material for: Perinatal use of triptans and other drugs for migraine—A nationwide drug utilization study
Source: PLoS One. 2021 Aug 23;16(8):e0256214. doi: 10.1371/journal.pone.0256214 (PMC8382165; doi:10.1371/journal.pone.0256214)
Supplement: S1 File — (DOCX) [file pone.0256214.s001.docx]

**Supplementary 1 (S1 Fig) Definition of exposure groups.**


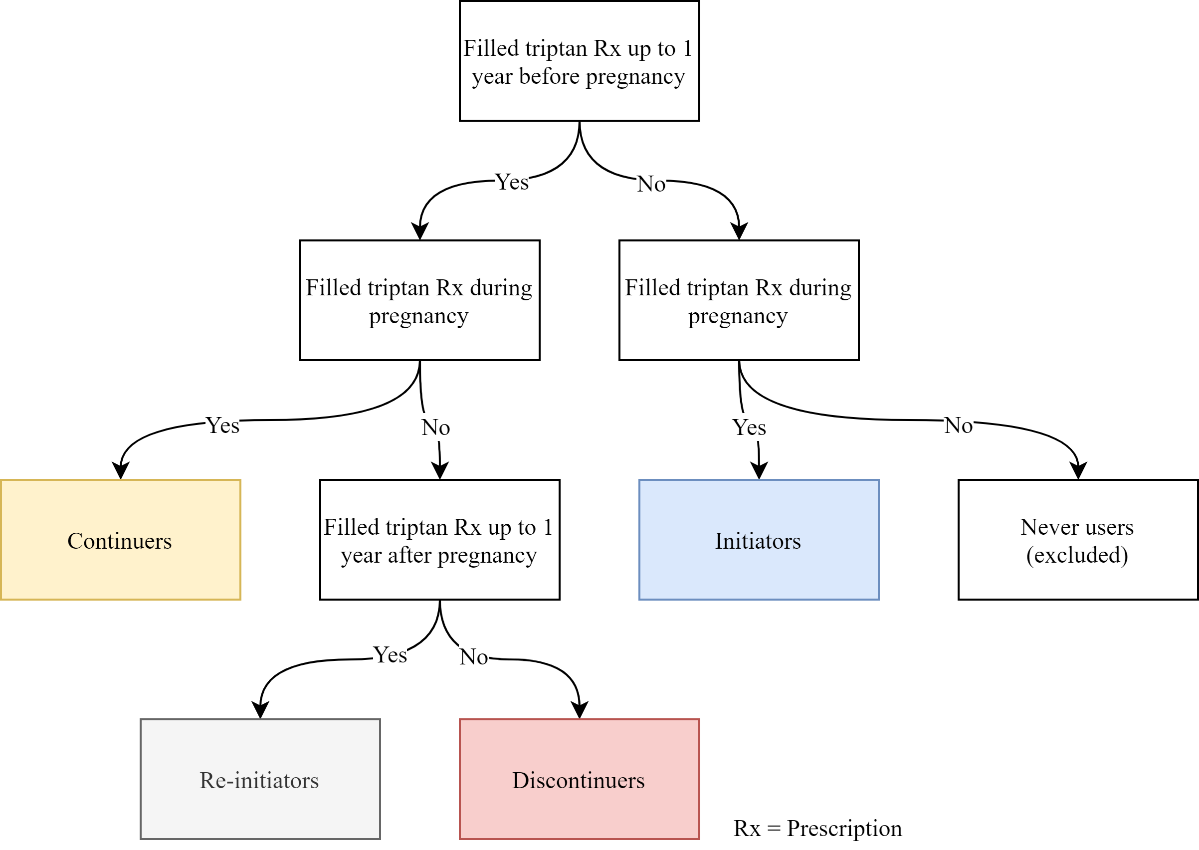


**Supplementary 2 (S2 Table) ATC codes, license status and DDDs of drugs included in the study.**

| Drugs | ATC codes | OTC status | | Licensed for use for migraine in Norway^(1)^ | DDDs ** |
| --- | --- | --- | --- | --- | --- |
| **Acute treatment – intermittent use*** |  |  | |  |  |
| **Triptans** |  |  |  |  |  |
| Sumatriptan | N02CC01 |  |  |  | 20 mg N  50 mg O  6 mg P  25 mg R |
| Naratriptan | N02CC02 |  |  |  | 2.5 mg O |
| Zolmitriptan | N02CC03 |  |  |  | 2.5mg N, O |
| Rizatriptan | N02CC04 |  |  |  | 10 mg O |
| Almotriptan | N02CC05 |  |  |  | 12.5 mg O |
| Eletriptan | N02CC06 |  |  |  | 40 mg O |
| Frovatriptan | N02CC07 |  |  |  | 2.5 mg O |
| **Analgesics** |  | | |  |  |
| Paracetamol | N02BE01 |  | Yes |  | 3 g O, P, R |
| Codeine and paracetamol | N02AJ06 |  |  |  | - |
| NSAIDs |  |  |  | No |  |
| Ibuprofen | M01AE01 |  | Yes |  | 1.2 g O, P, R |
| Diclofenac | M01AB05 |  | Yes |  | 0.1 g O, P, R |
| Naproxen | M01AE02 |  | Yes |  | 0.5 g O, R |
| Tolfenamic acid | M01AG02 |  |  |  | 0.3 g O, R |
| **Antinauseants** |  | | |  |  |
| Metoclopramide | A03FA01 |  |  |  | 30 mg O, P, R |
| Doxylamine | R06AA09 |  |  |  | 25 mg O |
| Meclozine | R06AE05 |  | Yes |  | 50 mg O, R |
| Prochlorperazine | N05AB04 |  |  |  | 0.1 g O, R  50 mg P |
| Promethazine | R06AD02 |  |  |  | 25 mg O, P, R |
| Serotonin (5HT_3_) antagonists | A04AA |  |  |  |  |
| Ondansetron | A04AA01 |  |  |  | 16 mg O, P, R |
| **Preventive treatment – continuous use*** | | | | Should be used only if the benefit of use outweighs the risk to the fetus |  |
| *First line* | |  |  |  |  |
| Metoprolol | C07AB02 |  |  |  | 0.15 g O, P |
| Propranolol | C07AA05 |  |  |  | 0.16 g O, P |
| *Second line* | |  |  |  |  |
| Amitriptyline | N06AA09 |  |  |  | 75 mg O, P |
| *Third line* | |  |  |  |  |
| Candesartan | C09CA06 |  |  |  | 8 mg O |
| Topiramate | N03AX11 |  |  |  | 0.3 g O |
| Valproic acid | N03AG01 |  |  |  | 1.5 g O, P, R |
| Botulinum toxin | M03AX01 |  |  |  | - |
| Clonidine | N02CX02 |  |  |  | 0.45 mg O |
| Lisinopril | C09AA03 |  |  |  | 10 mg O |
| Verapamil | C08DA01 |  |  |  | 0.24 g O, P |

*According to national clinical guidelines for the treatment of women with migraine (2, 3).

** DDDs (defined daily doses) for the main indication in adults; administration route = N (nasal), O (oral), P (parenteral), R (rectal) (4). Calcitonin gene-related peptide (CGRP) antagonists (N02CD) were first marketed for migraine prophylaxis in Norway in 2018, and thus no users were found.

| Medication for migraine | Moderate  n =21242 (%) | | | Severe  n = 1189 (%) | | | Very severe  n = 509 (%) | | |
| --- | --- | --- | --- | --- | --- | --- | --- | --- | --- |
|  | Pre | During | Post | Pre | During | Post | Pre | During | Post |
| **Analgesics** | 3286 (15.5) | 1313 (6.2) | 2477 (11.7) | 517 (43.5) | 241 (20.3) | 401 (33.7) | 308 (60.5) | 188 (36.9) | 265 (52.1) |
| Paracetamol | 740 (3.5) | 408 (1.9) | 727 (3.4) | 144 (12.1) | 89 (7.5) | 144 (12.1) | 102 (20.0) | 75 (14.7) | 112 (22.0) |
| Codeine and paracetamol | 1432 (6.7) | 854 (4.0) | 1027 (4.8) | 240 (20.2) | 166 (14.0) | 181 (15.2) | 155 (30.5) | 122 (24.0) | 132 (25.9) |
| NSAIDs | 2513 (11.8) | 362 (1.7) | 1763 (8.3) | 394 (33.1) | 65 (5.5) | 291 (24.5) | 233 (45.8) | 48 (9.4) | 181 (35.6) |
| **Antinauseants** | 440 (2.1) | 844 (4.0) | 320 (1.5) | 103 (8.7) | 119 (10.0) | 63 (5.3) | 68 (13.4) | 71 (14.0) | 41 (8.1) |
| **Preventive treatment** | No exposure by definition | | 290 (1.4) | 745 (62.7) | No exposure by definition | 153 (12.9) | 444 (87.2) | 509 (100.0) | 198 (38.9) |

**Supplementary 3 (S3 Table) Proportion of pregnancies with filled prescriptions for other drugs among migraine severity groups before, during, and after pregnancy, 2006–2017 (N = 22,940).**

**Moderate:** Use of triptans before and/or during pregnancy (excluding sumatriptan injection). **Severe:** Sumatriptan injection before and/or during pregnancy and/or migraine prophylaxis before but not during pregnancy. **Very severe:** Migraine prophylaxis in pregnancy.

**Supplementary 4 (S4 Table) Sensitivity analysis illustrating the proportion of pregnancies with filled prescriptions for other medications during pregnancy among exposure groups, excluding women with a history of epilepsy or chronic hypertension*, 2006–2017 (N = 22,476).**

| Medication for migraine | Triptan continuers  n = 4490 (%) | Triptan discontinuers  n = 12,229 (%) | Triptan initiators  n = 1813 (%) | Triptan re-initiators  n = 3945 (%) |
| --- | --- | --- | --- | --- |
| **Acute treatment** | 724 (16.1) | 1023 (8.4) | 236 (13.0) | 428 (10.9) |
| Analgesics | 574 (12.8) | 632 (5.2) | 190 (10.3) | 295 (7.5) |
| Paracetamol | 179 (4.0) | 213 (1.7) | 66 (3.6) | 96 (2.4) |
| Codeine and paracetamol | 385 (8.6) | 397 (3.3) | 114 (6.2) | 210 (5.3) |
| NSAIDs | 169 (3.8) | 170 (1.4) | 69 (3.7) | 54 (1.4) |
| Antinauseants | 254 (5.7) | 480 (3.9) | 88 (4.6) | 185 (4.7) |
| **Preventive treatment** | 203 (4.5) | 147 (1.2) | 33 (1.8) | 89 (2.39 |

*In total, 464 women had a history of epilepsy or chronic hypertension before pregnancy, as recorded in the medical birth charts, and were excluded from the sensitivity analysis.

| Parameters | | **Discontinuers (n = 11302)** | | | **Continuers (n = 3600)** | | | **Initiators (n = 1642)** | | | **Re-initiators (n = 3118)** | |
| --- | --- | --- | --- | --- | --- | --- | --- | --- | --- | --- | --- | --- |
|  | **Pre** | | **Pre** | **During** | | **Post** | **During** | | **Post** | **Pre** | | **Post** |
| Extended use of triptans^*^,  n (%) | **80 (0.7)** | | **417 (11.6)** | **T1: 247(6.9)**  **T2: 93 (2.6)**  **T3: 57 (1.6)** | | **193 (5.4)** | **T1: 11 (0.7)**  **T2: 5 (0.3)**  **T3: 7 (0.4)** | | **<5** | **102 (3.3)** | | **38 (1.2)** |
| Cumulative DDDs,  median (IQR) | **12 (6–24)** | | **45 (18–101)** | **T1: 15 (6–24)**  **T2: 12 (6–24)**  **T3: 18 (12–30)** | | **36 (18–90)** | **T1: 6 (6–12)**  **T2: 6 (6–12)**  **T3: 6 (6–12)** | | **12 (6–24)** | **24 (12–54)** | | **18 (10–36)** |
| Rx filled  Mean (SD) | **1.9 (1.7)** | | **5.4 (5.4)** | **1.9 (2.3)** | | **2.5 (4.4)** | **1.3 (0.9)** | | **0.4 (1.4)** | **3.3 (3.0)** | | **2.2 (2.1)** |

**Supplementary 5 (S5 Table) Drug utilization parameters for triptan use among exposure groups during and after pregnancy in a population with one random pregnancy per woman, 2006–2017 (n =19,669).**

**Supplementary 6 (S6 Table) Classification of migraine severity according to the triptan exposure groups in a population with one random pregnancy per woman, (n =19,669).**

| Exposure groups  n = 19,669 (100%) | Moderate migraine  n = 18,294 (93.0%) | Severe migraine  n = 969 (4.9%) | Very severe migraine  n = 406 (2.1%) |
| --- | --- | --- | --- |
| Triptan continuers (18.3) | 3146 (17.2) | 296 (30.6) | 158 (38.9) |
| Triptan discontinuers (57.5) | 10,729(58.7) | 429(44.3) | 144 (35.5) |
| Triptan initiators (8.4) | 1583 (8.7) | 35 (3.6) | 31 (7.6) |
| Triptan re-initiators (18.0) | 2836 (15.5) | 209 (21.6) | 73 (18.0) |
| Total | 18,294 (100.0) | 969 (100.0) | 406 (100.0) |

Please see Table 1 for the definition of migraine severity.

**References**

1. Felleskatalogen. Felleskatalogen 2020 [Available from: <https://www.felleskatalogen.no/medisin/;jsessionid=5AFg3n1wfqFUi4gMyPr4XLpz_acOuNm1f3Wznmt3.fkweb-live-web-cluster-fkweb-live-4>.

2. Danielsson K, Bjørk M, Sveberg L, Borthen I, Torkildsen Ø, Holmøy T, et al. Nevrologiske sykdommer i svangerskapet Norsk gynekologisk-forening: legeforeningen.no; 2020 [Available from: <https://www.legeforeningen.no/foreningsledd/fagmed/norsk-gynekologisk-forening/veiledere/veileder-i-fodselshjelp/nevrologiske-sykdommer-i-svangerskapet/>.

3. Evers S, Áfra J, Frese A, Goadsby PJ, Linde M, May A, et al. EFNS guideline on the drug treatment of migraine – revised report of an EFNS task force. European Journal of Neurology. 2009;16(9):968-81.

4. Methodology WCCfDS. ATC/DDD Index 2017. Oslo (NOR). 2019.
